# Effects of Dietary Supplementation with Three Different Probiotics on Growth Performance, Antioxidant Capacity, and Intestinal Microbiota in Grass Carp (*Ctenopharyngodon idella*)

**DOI:** 10.3390/microorganisms13061222

**Published:** 2025-05-27

**Authors:** Wanjia Zhu, Yi Yi, Zhiwei Zou, Haipeng Li, Ting Liang, Qianhe Shi, Liwei Liu, Jianmei Su

**Affiliations:** 1 Hubei Key Laboratory of Regional Development and Environmental Response, Faculty of Resources and Environmental Science, Hubei University, Wuhan 430062, China; 202321108012137@stu.hubu.edu.cn (W.Z.); 202421108012161@stu.hubu.edu.cn (Z.Z.); 202231108031008@stu.hubu.edu.cn (H.L.); 202231108031004@stu.hubu.edu.cn (T.L.); yiy@alphafeed.com (Q.S.); 2 College of Fisheries, Chinese Perch Research Center, Engineering Research Center of Green Development for Conventional Aquatic Biological Industry in the Yangtze River Economic Belt, Ministry of Education, Huazhong Agricultural University, No. 1, Shizishan Street, Hongshan District, Wuhan 430070, China; 202421108012111@stu.hubu.edu.cn

**Keywords:** *Bacillus subtilis*, *Clostridium butyricum*, *Enterococcus faecalis*, antioxidant ability, gut microbiota

## Abstract

The growing demand for sustainable aquaculture has intensified research on probiotics as antibiotic alternatives. This study aims to evaluate the effects of three probiotic supplements—1 × 10^10^ CFU/g of *Bacillus subtilis* (BS), *Clostridium butyricum* (CB), or *Enterococcus faecalis* (EF)—on growth performance, antioxidant capacity, intestinal structure, and gut microbiota in grass carp (*Ctenopharyngodon idella*; initial body weight: 42.52 ± 4.17 g) for 28 d. Compared to the non-supplemented (NC) control group, all probiotic-supplemented groups significantly enhanced final body weight, weight gain rate, specific growth rate, and crude protein content, and reduced feed conversion ratio (*p* < 0.05). Probiotic supplementation upregulated the intestinal *ctrb1* gene expression and increased villus length. Serum superoxide dismutase (SOD) and catalase activity were elevated in the BS group, whereas only SOD was increased in the CB group (*p* < 0.05). Gut microbiota analysis revealed reduced Proteobacteria abundance in all probiotic-supplemented groups. Compared with the NC group, the BS group enriched Bacteroidetes and *Prevotella_7*, while the CB group promoted the abundance of Actinobacteria, *Lactobacillus,* and *Clostridium_sensu_stricto_1*. The EF group increased the abundance of Fusobacteria, *Cetobacterium*, and *Bacteroides* (*p* < 0.05). These findings demonstrate that dietary supplementation with probiotics enhances growth performance by modulating antioxidant responses, intestinal morphology, and microbial community balance.

## 1. Introduction

In recent years, feed demand has driven a surge due to the expansion of intensive aquaculture practices [1]. With the increasing price of feed raw materials, cheap plant-based proteins have been widely used in large quantities in feeds, which not only reduce feed palatability and its utilization efficiency but also weaken the immune function of fish, causing tissue damage and infections of fish [2,3,4]. To mitigate fish disease outbreaks, antibiotics have been used routinely in aquaculture. However, prolonged antibiotic overuse has led to problems including drug residue, antibiotic resistance, and decreased fish immunity [5,6]. Consequently, the development and application of antibiotic alternatives with no side effects on the environment are classified as a critical priority for sustainable and eco-friendly aquaculture development.

Probiotics, defined as beneficial microorganisms that can be colonized in the fish gut or improve aquaculture water quality, play a positive role in growth performance, nutrient absorption, energy metabolism, and immune response of fish [7,8,9]. Probiotics can alleviate the adverse effects of high-density farming and the side effects and pollution caused by antibiotics and other drugs, thereby showing great application prospects [10,11]. Currently, prevalent probiotic strains, including *Bacillus*-like, lactic acid bacteria-like, and fungal-like probiotics, have been successfully applied in the feed of grass carp (*Ctenopharyngodon idella*), common carp (*Cyprinus carpio* L.), South American catfish (*Rhamdia quelen*), Nile tilapia (*Oreochromis niloticus*), and largemouth bass (*Micropterus salmoides*) [1,12,13,14,15,16].

*Bacillus subtilis* stands out for its production of abundant extracellular enzyme systems and participation in degrading harmful substances such as ammonia nitrogen and nitrite, thereby improving aquaculture water quality [12,17]. *B. subtilis* increases the digestive enzyme activity in aquatic animals, modulates hepatic lipid metabolism, reduces fat deposition, and consequently improves the growth performance and serum biochemical profiles of aquatic animals [18,19,20]. For example, *B*. *subtilis* at 10^8^ or 10^9^ CFU/g can improve intestinal protease activity in Nile tilapia (*O*. *niloticus*) or Yellow River carp (*C. carpio* L.), respectively [21,22]. *B. subtilis* can also compete with pathogens for intestinal adhesion sites and nutrients by producing antibiotics and bacteriocins. Therefore, *B. subtilis* inhibits the adherence of pathogens to fish intestinal epithelial cells, resulting in the enrichment of anaerobic beneficial bacteria. Dietary supplementation of *B. subtilis* has been reported to restructure the intestinal microbiota, repair tissue damage, and enhance the intestinal immune function, thereby boosting fish growth [23,24].

*Clostridium butyricum* and *Enterococcus faecalis* are widely recognized as short-chain fatty acid-producing bacteria that can enhance metabolism, improve intestinal morphology, optimize gut microbiota composition, and strengthen mucosal immunity in fish by producing short-chain fatty acids [25]. *C. butyricum* can ferment polysaccharides to generate butyric acid, which can prevent the colonization of enteropathogens by creating an acidic environment. It has been reported that *C. butyricum* has a significant inhibitory effect on pathogenic bacteria (e.g., *Enterobacter*), while it enriches beneficial bacteria such as *Bifidobacterium* and *Lactobacillus*. *C. butyricum* can also enhance the resistance of fish to pathogens such as *Streptococcus agalactiae* and *Carassius auratus herpesvirus*, thus reducing the mortality rate of fish [26,27].

*E. faecalis*, a type of lactic acid bacterium, is also an indigenous bacterium in the fish gut. It has been demonstrated that *E. faecalis* can synthesize L-type lactic acid through glycolytic metabolism, thereby facilitating the intestinal calcium absorption in fish. In addition, *E. faecalis* can secrete various enzymes (proteases, lipases, and amylases) to catalyze the macromolecule breakdown, thus improving the nutrient utilization efficiency of fish [28,29]. For instance, supplementation with *E*. *faecalis* or *Lactococcus lactis* at 10^8^ CFU/g enhanced protease activity in the intestine of snakehead fish (*Channa argus*) [30]. Earlier research has demonstrated that dietary supplementation with *E. faecalis* at 1 × 10^7^ CFU/g significantly elevated the weight gain rate, specific growth rate, and serum alkaline phosphatase (ALP) activity of tilapia (*O*. *niloticus*) [31]. It has also been reported that *E. faecalis* produces antimicrobials, such as bacteriocins and antimicrobial peptides, to specifically inhibit or kill pathogens, thereby maintaining intestinal microbial balance [32,33].

Numerous studies have documented the positive effects of probiotics on intestinal digestive enzyme activity in fish [21,22,34]. Chymotrypsin, a serine proteolytic enzyme that hydrolyzes the carboxyl side chains of aromatic amino acid residues, not only plays a critical role in protein digestion and energy metabolism in fish, but also serves as a key biomarker for evaluating nutritional status and growth efficiency of fish [34,35]. For instance, dietary supplementation with a synbiotic containing *Lactococcus lactis* significantly enhanced chymotrypsin activity in the intestine of Caspian roach (*Rutilus frisii kutum*), along with improvements in body weight gain and protein efficiency [36]. Similarly, the metabolite of *C. butyricum* also increased intestinal chymotrypsin activity in grass carp [37]. Chymotrypsin is synthesized in the pancreas as an inactive precursor, chymotrypsinogen, which is subsequently secreted into the intestine to be activated [35]. Consequently, the expression of the chymotrypsinogen B gene (*ctrb1*) directly influences chymotrypsin activity [38]. However, limited research has addressed how probiotics regulate *ctrb1* expression in fish. Therefore, investigating the effects of dietary probiotics on the *ctrb1* gene expression in grass carp is essential for understanding their role in modulating digestive capacity and nutritional status in aquaculture species.

Grass carp (*C. idella*) is the most prevalent freshwater farmed fish in China and is also listed of the four major fish in China because of its rapid growth, adaptability, wide food source, low protein requirement, and delicious meat flavor [39]. Although these probiotics have been extensively studied in various fish species, most research has focused on identifying optimal supplementation gradients for single strains [40] or evaluating the efficacy of individual and combined strains [30]. In contrast, limited studies have systematically compared the probiotic effects of *B*. *subtilis*, *C*. *butyricum*, and *E*. *faecalis* on grass carp at equivalent concentrations. Furthermore, previous investigations predominantly emphasized growth performance and immune responses in fish, while the specific mechanisms by which these probiotics improve intestinal digestion and modulate gut microbiota in grass carp remain poorly elucidated. To address these gaps, this study conducted a 28-day feeding trial using three experimental diets supplemented with *B. subtilis*, *C. butyricum*, and *E. faecalis* at 1 × 10^10^ CFU/g, respectively. We comprehensively analyzed and compared the effects of these probiotics on growth performance, digestive capacity, antioxidant activity, intestinal morphology, and gut microbiota in grass carp. Additionally, the regulatory impact of probiotic supplementation on intestinal *ctrb1* gene expression levels was investigated. These findings provide theoretical insights to optimize the application of these probiotics in aquaculture practices.

## 2. Materials and Methods

### 2.1. Preparation of Probiotic Additives and Experimental Diets

The experiment consisted of four groups: the control group (NC), the *B. subtilis* group (BS), the *C. butyricum* group (CB), and the *E. faecalis* group (EF), with three replicates per group. The feed in the NC group did not contain any probiotics, while the feeds in the BS, CB, and EF groups contained *B. subtilis*, *C. butyricum*, and *E. faecalis* were obtained from Wuhan SunHY Biology Co., Ltd. (Wuhan, China). All feed ingredients except probiotics were weighed according to the basic diet formula (Table 1) and thoroughly mixed in a mixer. Oil was then added and mixed for 5 min. Subsequently, deionized water (400 mL/kg of dry ingredient mixture) was added and mixed for another 5 min. The mixture was then ground and passed through an 80-mesh sieve. A pellet mill was used to form the mixture into 2 mm pellets.

Following granulation, three probiotic agents were sprayed onto the diet at specified ratios, air-dried, and stored in sealed plastic bags at −20 °C. Prior to the experiment, feed samples were analyzed by Wuhan SunHY Biology Co., Ltd. (Wuhan, China) to quantify probiotic concentrations using the dilution plate counting method. The final concentrations of *B. subtilis* (BS), *C. butyricum* (CB), and *E. faecalis* (EF) in diets were confirmed as 1 × 10^10^ CFU/g. To maintain bacterial viability, diets were stored at −20 °C throughout the 28-day trial. Weekly monitoring confirmed stable probiotic viability in the probiotic-supplemented feed (0.97 × 10^10^–0.99 × 10^10^ CFU/g).

### 2.2. Experimental Fish and Design

Grass carp were purchased from Wuhan Baishazhou Aquatic Market (Wuhan, China). After a 14-day acclimatization period, 240 grass carp of uniform size, robust health, and no injuries were selected. The grass carp, with an initial body weight (IBW) of 42.59 ± 0.46 g, were randomly distributed into 12 experimental tanks (300 L in volume, 1 m in diameter), with three tanks per group and a stocking density of 20 fish per tank. Before the start of the experiment, the fish were fasted for 24 h, and their initial weights were measured. The 28-day breeding experiment was conducted at the Aquaculture Experiment Base of the College of Fisheries, Huazhong Agricultural University (Wuhan, China). During the experiment, the fish were fed to satiation twice daily (at 8:30 and 16:30) with a feeding amount not exceeding 3% of the grass carp’s body weight. Two hours after feeding, feces and leftover feed were siphoned off, dried at 60 °C for 12 h until constant weight, and then weighed. Daily feed intake and mortality were recorded. The water was changed twice daily, with a daily water exchange rate of 40% to 60% of the total volume, and continuous aeration was provided for 24 h to ensure water quality requirements. During the experiment, the water quality was monitored and adjusted as follows: water temperature was maintained at 12–15 °C, dissolved oxygen at 7 ± 0.45 mg/L, pH at 7.5 ± 0.3, and the photoperiod was set to the natural light cycle. Before the end of the experiment, the fish were fasted for 24 h, weighed, and counted. Growth performance indices, including feed intake rate (FR), weight gain rate (WGR), specific growth rate (SGR), feed conversion ratio (FCR), and protein efficiency ratio (PER), were calculated.

### 2.3. Sample Collection

After 28 days of cultivation, all grass carp were collected from each tank. The fish were anesthetized with MS-222 (10 mg/L) for counting and weighing to determine growth performance. Three fish were randomly selected from each tank, quickly frozen in liquid nitrogen, and then transferred to a −20 °C freezer for crude protein analysis. Three additional fish were randomly selected from each tank for tail vein blood collection to determine serum antioxidant enzyme activities. Furthermore, two fish from each tank were anesthetized and rapidly dissected on ice. Intestinal tissue samples were collected, frozen in liquid nitrogen, and stored at −80 °C for quantitative gene expression analysis. Another portion of the intestinal samples was fixed in a 4 °C fixative for intestinal morphology analysis. Before intestinal microbiota collection, the grass carp were subjected to a 12-h fasting period. Two fish from each tank were anesthetized, and their body surface was rinsed with 0.9% saline solution. The intestines were rapidly removed using sterile surgical tools and placed in sterile Petri dishes. The intestines were longitudinally opened, the intestinal fat tissue was removed, and the intestinal contents were quickly scraped and frozen in liquid nitrogen, then transferred to −80 °C for subsequent intestinal microbiota analysis.

### 2.4. Calculation of Growth Performance

The survival rate and growth performance indices of grass carp (survival rate, feed rate, specific growth rate, and feed conversion ratio) were calculated using the following formulas:

Specific growth rate (SGR, %/d) = 100 × (Ln final body weight − Ln initial body weight)/day(1)

Survival rate (SR, %) = 100 × (total fish − dead fish)/total fish(2)

Weight gain rate (WGR, %) = 100 × (final body weight − initial body weight)/initial body weight(3)

Feed rate (FR, %) = 100 × feed intake/(final body weight + initial body weight)/2 × day(4)

Feed conversion ratio (FCR) = feed intake/weight gain(5)

Protein efficiency ratio (PER) = weight gain/protein intake(6)

### 2.5. Analysis of Crude Protein Content in Fish Body

The frozen samples stored at low temperatures were retrieved, and the crude protein content in the fish body was determined using the Kjeldahl method as described in previous studies [15].

### 2.6. Determination of Serum Antioxidant Enzyme Activity

At the end of the experiment, the fish were deprived of food for 24 h. Two grass carp were randomly selected from each tank, anesthetized, and blood was collected from the tail vein using sterile syringes (2 mL). Blood samples were left to stand overnight at 4 °C and then centrifuged at 5000 r/min for 10 min. The upper serum layer was collected, aliquoted into centrifuge tubes, and stored at −80 °C. The activities of total superoxide dismutase (SOD) and catalase (CAT) in the serum were measured using the Total Superoxide Dismutase Assay Kit and the Catalase Assay Kit (Nanjing Jiancheng Bioengineering Research Institute, Nanjing, China), respectively, following the manufacturer’s instructions.

### 2.7. Quantitative Gene Expression Analysis

Total RNA was extracted from fish intestinal tissue using the RNAiso Plus Kit (Takara, Dalian, China). RNA purity and concentration were measured using a NanoDrop 2000 spectrophotometer (Thermo Scientific, Waltham, MA, USA), and RNA quality and integrity were assessed by 1.0% agarose gel electrophoresis. cDNA was synthesized from the RNA using the Evo M-MLV Reverse Transcription Premix Kit (Aikuri Biotechnology, Changsha, China) and stored at −20 °C.

Chymotrypsin is a critical digestive enzyme for protein degradation, and the chymotrypsinogen B1 gene (*ctrb1*) encodes the precursor of chymotrypsin [35]. Primers specific to the *ctrb1* gene were designed using Primer Premier 6.0 software: forward primer *ctrb1-F* (GGGGCCTGACCAGGTACAAT) and reverse primer *ctrb1-R* (CCAGAGGACCACCAGAATCAC), synthesized by Sangon Biotech Co., Ltd. (Shanghai, China). Quantitative real-time PCR (qRT-PCR) was performed using the Roche Light Cycler 480^®^ real-time PCR instrument (Roche, Indianapolis, IN, USA) with cDNA as the template. The reaction system (20 μL) consisted of 1 μL cDNA, 0.4 μL *ctrb1-F*, 0.4 μL *ctrb1-R*, 10 μL 2 × SYBR Green qPCR Mix, and 8.2 μL diethylpyrocarbonate water (Novozyme, Nanjing, China). The reaction program was as follows: 95 °C for 30 s, followed by 40 cycles of 95 °C for 5 s and 60 °C for 30 s. Each reaction was performed in triplicate. The relative expression levels of the *ctrb1* gene in the intestines of grass carp from different groups were analyzed using the 2^−ΔΔCT^ method, with *β-actin* as the reference gene for normalization [41].

### 2.8. Intestinal Morphological Analysis

At the end of the cultivation experiment, three grass carp were collected from each tank. After being rinsed with 0.9% saline solution and distilled water, the fish were dissected. Intestinal tissue samples (approximately 4 mm in length) were fixed in 4% paraformaldehyde fixative (Wuhan Service, Wuhan, China) at 4 °C. The samples were then subjected to graded ethanol dehydration, xylene cleaning, paraffin embedding, and sectioning (5 μm thickness), followed by hematoxylin-eosin (HE) staining (Wuhan Sevicebio Technology Co., Ltd., Wuhan, China). Morphological observations of the intestinal samples were conducted using Zeiss Axio Imager A2 fluorescent microscope (Zeiss, Oberkochen, Germany), and photographs were taken at 40× magnification under identical background lighting. The lengths of intestinal villi were measured using Image-Pro Plus 6.0 software [42,43].

### 2.9. Intestinal Microbiota Profiling

Intestinal contents from four randomly selected fish per group were stored at −80 °C. The samples were sent to Biomarker Technologies Co., Ltd. (Beijing, China) for 16S rRNA high-throughput sequencing. Bacterial DNA was extracted from the intestinal samples using the E.Z.N.A.^®^ Soil DNA Kit (Omega, Norcross, GA, USA), and the integrity and quality of the DNA samples were assessed by 1% agarose gel electrophoresis. The purity and concentration of the DNA were determined using a NanoDrop 2000 spectrophotometer (Thermo Scientific, Norcross, GA, USA) [9].

PCR amplification of the V3-V4 variable regions of the 16S rRNA gene was performed using the forward primer 338F (5′-ACTCCTACGGGAGGCAGCAG-3′) and reverse primer 806R (5′-GGACTACHVGGGTWTCTAAT-3′) on a T100 Thermal Cycler (BIO-RAD, Hercules, CA, USA). The PCR products were purified, quantified, and normalized to create sequencing libraries. Qualified libraries were sequenced using the Illumina HiSeq 2500 (Illumina, San Diego, CA, USA) with paired-end sequencing. The original data were assembled using FLASH (version 1.2.11), and the assembled sequences were quality filtered (Trimmomatic, version 0.33) and chimerism was removed (UCHIME, version 8.1) to obtain high-quality tag sequences [44].

Effective sequences were analyzed using Usearch 10.0 software since operational taxonomic units (OTUs) have been widely adopted in fish gut microbiota studies [27,30,45]. OTUs were clustered at a sequence similarity of 97% and were subjected to rarefaction processing. The Good’s coverage in all groups remained at 99.09%, confirming sufficient sequencing depth to reliably represent microbial community diversity. Taxonomic annotations of OTUs were performed using the RDP Classifier 2.2 software based on the Silva 16S rRNA gene database (version 132, http://www.arb-silva.de, accessed on 18 November 2019). The microbial community composition of each sample was analyzed at different taxonomic levels (phylum, class, order, family, genus, and species), and diversity indices such as Shannon, Simpson, Chao, and Ace were calculated [36]. To mitigate the influence of sequencing depth on α- and β-diversity analyses, all samples were subjected to rarefaction processing. Post-rarefaction, the average sequence coverage (Good’s coverage) remained at 99.09%, confirming sufficient sequencing depth to reliably represent microbial community diversity.

### 2.10. Statistical Analysis

Statistical analyses were performed using SPSS 25.0 software, and graphs were created using GraphPad Prism 9.0 software. Numerical data are expressed as mean ± standard error (mean ± SE). One-way ANOVA and Duncan’s test were used for statistical analysis. The significance threshold was set at *p* < 0.05. Different letters or asterisks (*) indicate significant differences between groups (*p* < 0.05), while identical letters or no markings indicate no significant differences between groups (*p* > 0.05).

## 3. Results

### 3.1. Effects of Dietary Probiotic Supplementation on Growth Performance of Grass Carp

The effects of different probiotics on the growth performance of grass carp are presented in Table 2. Compared to the NC group, the SR in the BS group showed no significant difference, while the SR in the CB and EF groups was significantly higher (*p* < 0.05). The FBW, WGR, FR, and SGR of grass carp in the BS, CB, and EF groups were significantly higher than those in the NC group (*p* < 0.05), while the FCR was significantly lower (*p* < 0.05). For PER, the BS group showed an increasing trend compared to the NC group, but the difference was not significant (*p* > 0.05). However, the PER in the CB and EF groups was significantly higher than that in the NC and BS groups (*p* < 0.05).

### 3.2. Effects of Probiotics in Feed on Crude Protein Content of Grass Carp

**Figure 1:**
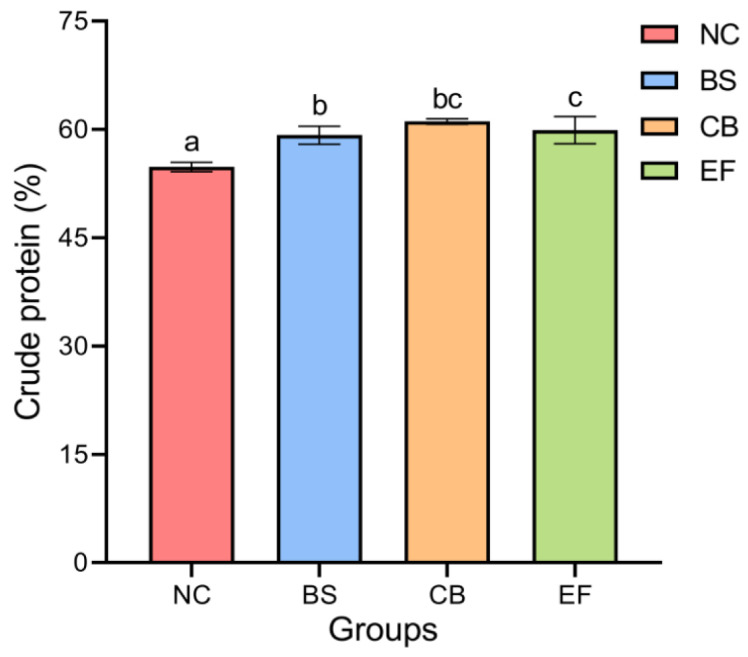
The crude protein contents of grass carp in different probiotic-supplemented groups and the control group. Bars marked with different letters represent statistically significant differences (*p* < 0.05).

As shown in Figure 1, the crude protein content in the BS, CB, and EF groups was 59.20 ± 1.24%, 61.11 ± 0.38%, and 57.37 ± 0.39%, respectively, all of which were significantly higher than that in the NC group (54.79 ± 0.67%) (*p* < 0.05). This indicates that the addition of three types of probiotics (*B. subtilis*, *C. butyricum*, and *E. faecalis*) in feed can significantly increase the crude protein content of grass carp.

### 3.3. Effects of Dietary Probiotic Supplementation on Serum Antioxidant Enzyme Activity in Grass Carp

**Figure 2:**
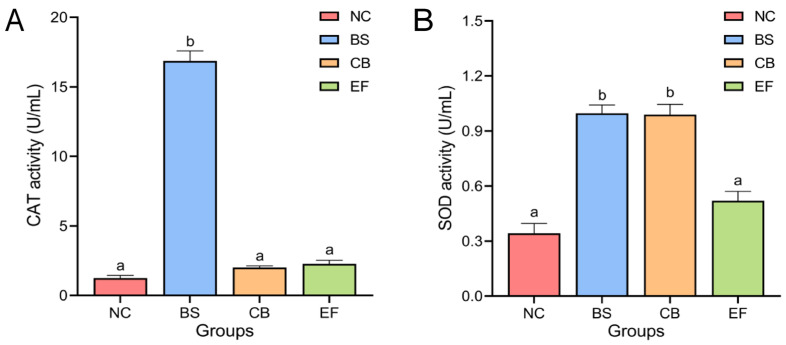
Activity of serum antioxidant enzymes (**A**) CAT and SOD (**B**) in grass carp from different probiotic-supplemented groups and the control group (U/mL). Bars marked with different letters represent statistically significant differences (*p* < 0.05).

The effects of different probiotic additions on the activity of antioxidant enzymes CAT and SOD in grass carp serum are shown in Figure 2. The serum CAT activity of grass carp in the BS group (16.87 ± 0.72 U/mL) was significantly higher than in the NC group (1.25 ± 0.19 U/mL). The serum CAT activity of grass carp in the CB group (2.01 ± 0.12 U/mL) and EF group (2.27 ± 0.25 U/mL) was higher than that in the NC group (1.25 ± 0.19 U/mL), but there was no significant difference (*p* > 0.05).

Serum SOD activity in the BS group (1.00 ± 0.05 U/mL) and the CB group (0.99 ± 0.06 U/mL) were significantly higher than that of the serum SOD activity of the NC group (0.34 ± 0.05 U/mL) (*p* < 0.05), whereas the difference between the serum SOD activity of the EF group (0.52 ± 0.05 U/mL) and that of the NC group was not significant (*p* > 0.05).

### 3.4. Effects of Dietary Probiotic Supplementation on Chymotrypsinogen B1 Gene Expression in the Intestine of Grass Carp

**Figure 3:**
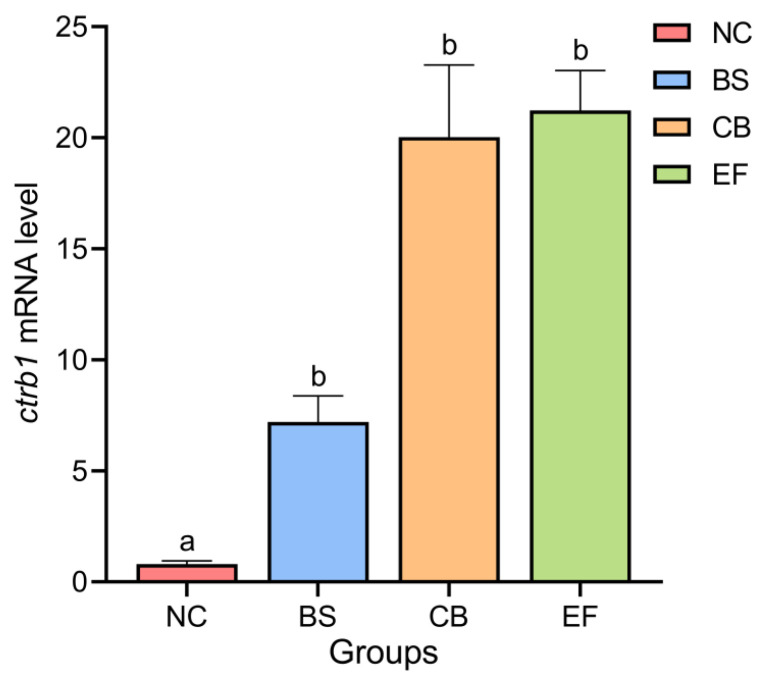
Relative expression levels of the *ctrb1* gene in the intestines of grass carp from different probiotic-supplemented groups and the control group. Bars marked with different letters represent statistically significant differences (*p* < 0.05).

As shown in Figure 3, the relative expression levels of the Chymotrypsinogen B1 gene (*ctrb1*) in the intestines of the NC, BS, CB, and EF groups were 0.79 ± 0.15, 7.20 ± 1.17, 20.03 ± 3.25, and 21.23 ± 1.80, respectively. Compared to the NC group, the relative expression levels of the *ctrb1* gene in the intestines of the BS, CB, and EF groups were significantly higher (*p* < 0.05).

### 3.5. Effects of Dietary Probiotic Supplementation on Intestinal Histomorphology of Grass Carp

**Figure 4:**
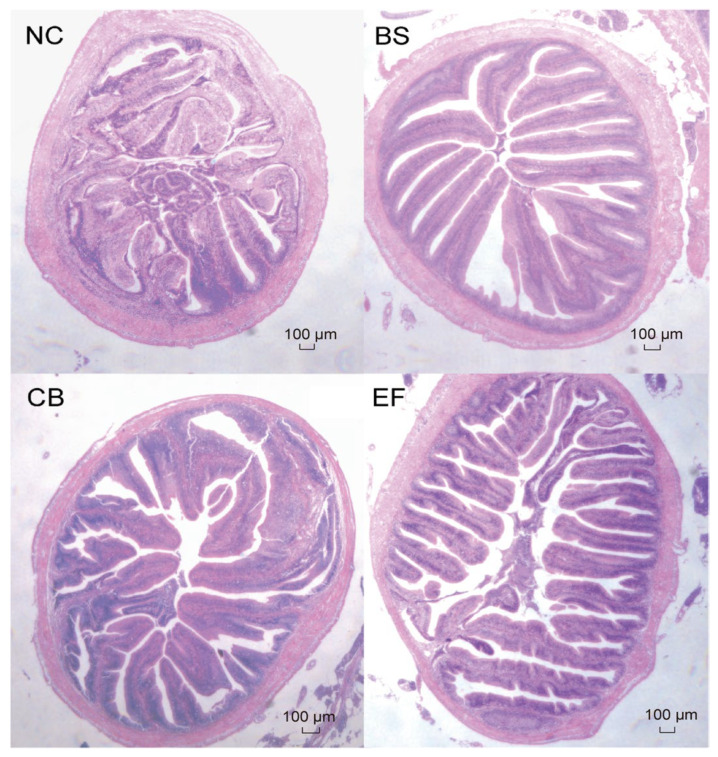
Microstructure of grass carp intestines from different groups (scale bar = 100 µm, original magnification ×400).

As shown in Figure 4, the intestinal epithelial cells of grass carp in all groups were structurally intact, and the distribution of intestinal villi was relatively uniform, indicating that the morphology and structure of grass carp in all groups were normal.

As shown in Table 3, compared to the NC group, the villus height (VH) in the BS, CB, and EF groups was significantly higher (*p* > 0.05). However, there were no significant differences in crypt depth (CD) and villus height/crypt depth ratio (V/C) among the groups (*p* > 0.05), except that the V/C in the BS group was significantly higher than that in the NC group (*p* < 0.05).

### 3.6. Effects of Dietary Probiotic Supplementation on Intestinal Microbiota of Grass Carp

The effects of different probiotics on the diversity of gut microbial communities in grass carp are presented in Table 4. The Chao, Shannon, and Simpson indices in the BS and EF groups were significantly lower than those in the NC group (*p* < 0.05), while the Ace index showed no significant difference (*p* > 0.05). The Chao index in the CB group was significantly higher than that in the NC group (*p* < 0.05), while the Ace, Shannon, and Simpson indices showed no significant differences (*p* > 0.05).

**Figure 5:**
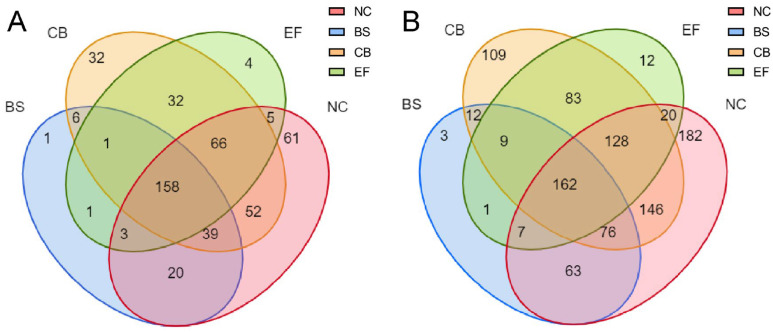
Venn diagrams of gut microbiota in grass carp from different probiotic-supplemented groups and the control group on the OTU level (**A**) and genus level (**B**).

Venn diagram analysis of all grass carp gut microbial communities at the OTU and genus levels (Figure 5) showed that the NC had the highest number of unique OTUs (61), the BS group had one unique OTU, the CB had 32 unique OTUs, the EF had four unique OTUs, and the probiotic-added group and the control group shared 158 OTUs.

At the genus level, the NC had the highest number of unique taxa (182), the BS group had three, the CB had 109, the EF had 12, and the probiotic-added group shared 162 taxa with the control group. At different taxonomic levels, the BS group had fewer unique taxa than the remaining groups, suggesting that the microbial richness in its group was lower, but its dominant populations were more prominent.

**Figure 6:**
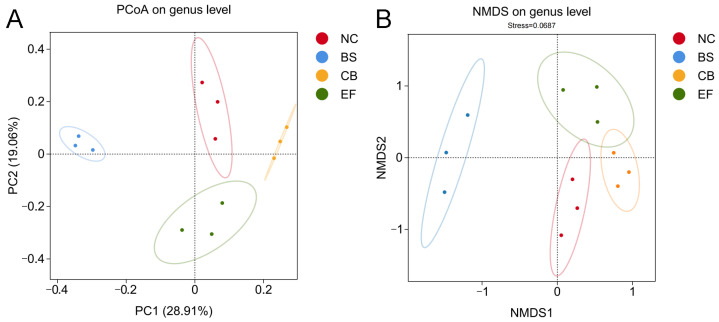
β-diversity analysis of gut microbiota of grass carp in different probiotic-supplemented groups and control group. (**A**) Principal coordinate analysis (PCoA) plot based on the binary_jaccard distance matrix; (**B**) Non-metric multidimensional scaling (NMDS) plot based on the binary_jaccard distance matrix.

Principal coordinate analysis (PCoA) and non-metric multidimensional scaling (NMDS) based on genus-level data (Figure 6) showed that the probiotic-supplemented groups (BS, CB, EF) were distant from the NC group, while samples within each probiotic group were closely clustered. This indicates that the gut microbiota structure of grass carp in the probiotic-supplemented groups differed significantly from those in the NC group, suggesting that the three types of probiotics in the feed altered the gut microbiota composition of grass carp.

**Figure 7:**
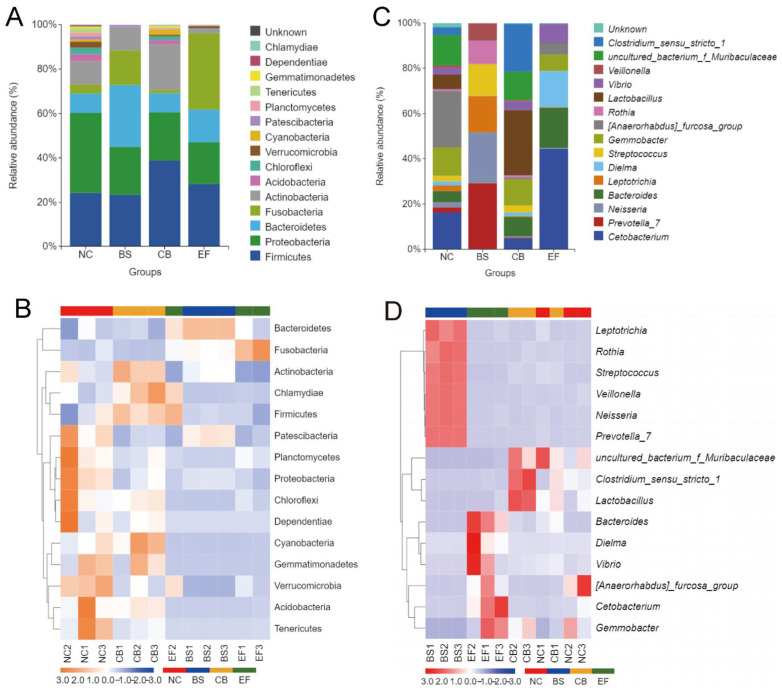
Composition of gut microbiota of grass carp in different probiotic-supplemented groups and the control group. (**A**) Histograms on phylum level; (**B**) Heatmaps on phylum level; (**C**) Histograms on genus level; (**D**) Heatmaps of gut community on genus level.

Analysis of the relative abundance of gut microbiota at the phylum level in grass carp from different probiotic-supplemented groups is shown in Figure 7A,B. The four groups had similar major phyla, including Firmicutes, Proteobacteria, Bacteroidetes, Fusobacteria, Actinobacteria, and Acidobacteria, but with differences in relative abundance. The relative abundance of Proteobacteria in the BS, CB, and EF groups was 21.76%, 21.81%, and 18.96%, respectively, all significantly lower than in the NC group (36.12%) (*p* < 0.05). The relative abundance of Verrucomicrobia in the BS, CB, and EF groups was below 1%, also significantly lower than that in the NC group (2.63%) (*p* < 0.05). The Bacteroidetes relative abundance in the BS group (27.75%) was significantly higher than in the NC group (8.58%) (*p* < 0.05), while the Acidobacteria relative abundance (0.03%) was significantly lower than in the NC group (3.19%) (*p* < 0.05). The Actinobacteria and Cyanobacteria relative abundances in the CB group were 20.33% and 2.26%, respectively, both significantly higher than in the NC group (10.46% and 0.94%) (*p* < 0.05). The Fusobacteria relative abundance in the EF group (34.19%) was significantly higher than that in the NC group (4.08%) (*p* < 0.05), while the Actinobacteria relative abundance (1.85%) was significantly lower than in the NC group (10.46%) (*p* < 0.05). Additionally, the relative abundance of Patescibacteria in the CB and EF groups (0.24% and 0.10%, respectively) was significantly lower than in the NC group (1.39%) (*p* < 0.05).

At the genus level, the dominant genera in the gut microbiota of grass carp were *Cetobacterium*, *Gemmobacter*, and *Bacteroides*, but their relative abundances varied among the groups. In the BS group, the relative abundance of *Prevotella_7*, *Neisseria*, *Leptotrichia*, and *Streptococcus* was significantly higher than in the other groups (*p* < 0.05). In the CB group, the relative abundance of *Lactobacillus* and *Clostridium_sensu_stricto_1* was significantly higher than in the other groups (*p* < 0.05). In the EF group, the relative abundance of *Cetobacterium* and *Bacteroides* was significantly higher than in the other groups (*p* < 0.05).

**Figure 8:**
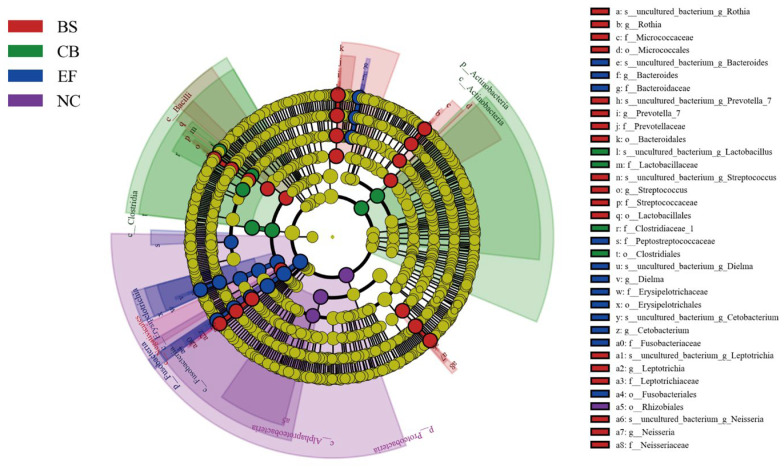
LEfSe analysis of microbial communities from phylum to genus level (LDA = 4.5) in probiotic-supplemented groups and the control group.

Linear discriminant analysis effect size (LEfSe) analysis based on one-to-one comparisons of gut microbiota from phylum to genus levels is shown in Figure 8. In the BS group, the relative abundance of *Prevotella_7*, *Streptococcus*, *Prevotellaceae*, *Bacteroidales*, and *Lactobacillales* was significantly higher than in the NC group (*p* < 0.05). In the CB group, the relative abundance of *Lactobacillaceae*, *Clostridiaceae_1*, and *Clostridiales* was significantly higher than in the other groups (*p* < 0.05). In the EF group, the relative abundance of *Bacteroides*, *Cetobacterium*, *Bacteroidaceae*, *Fusobacteriaceae*, and *Fusobacteriales* was significantly higher than in the NC group (*p* < 0.05), consistent with the results shown in Figure 7.

**Figure 9:**
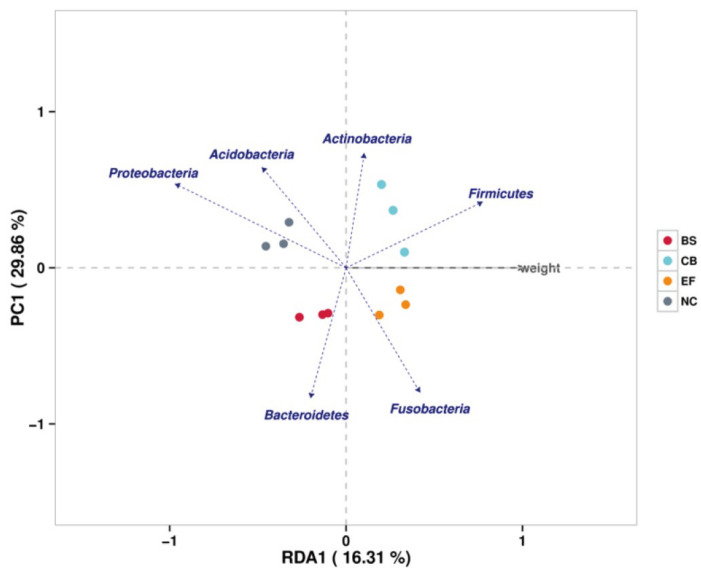
Redundancy analysis (RDA) correlating the abundance of intestinal microbiota with the body weight of grass carp in different probiotic-supplemented groups and the control group.

Redundancy analysis (RDA) showed that phyla such as Firmicutes, Fusobacteria, and Actinobacteria were positively correlated with increased fish weight, while Proteobacteria showed a negative correlation. This suggests that specific gut microbiota may play a role in promoting fish growth by influencing nutrient metabolism and energy utilization (Figure 9).

## 4. Discussion

### 4.1. Dietary Probiotic Supplementation Enhances Growth Performance in Grass Carp

The results of this study demonstrated that compared to the NC group, the BS group supplemented with *B. subtilis* (1 × 10^10^ CFU/g) showed no significant improvement in SR but exhibited significantly increased FBW, WGR, SGR, and crude protein content (*p* < 0.05), along with a significant reduction in FCR (*p* < 0.05). These findings align with previous studies on *B. subtilis* in Japanese eel (*A. japonica*) and Nile tilapia (*O*. *niloticus*). For instance, dietary supplementation of *B. subtilis* WB60 or *B. subtilis* HAINUP40 at 1.0 × 10^8^ CFU/g significantly improved FBW, FCR, PER, and SGR in fish, while also enhancing post-infection survival rates of *Vibrio anguillarum*-challenged Japanese eels [21,46].

In the present study, the CB and EF groups exhibited significantly higher SR, FW, WGR, SGR, PER, and crude protein content compared to the NC group (*p* < 0.05). The study of Wang et al. confirmed that grass carp (*C. idella*) fed with *Clostridium butyricum* metabolites had higher growth performance (FBW, WGR, and SGR) [39]. Similarly, *C. butyricum* has been reported to improve growth performance in largemouth bass (*M. salmoides*), Nile tilapia (*O. niloticus*), large yellow croaker (*Larimichthys crocea*), and yellow catfish (*Pelteobagrus fulvidraco*), with optimal supplementation levels ranging from 1.0 × 10^6^ to 1.5 × 10^8^ CFU/g [27,47,48,49]. Furthermore, dietary supplementation with *E. faecalis* W24 or *E. faecium* L6 at 1.0 × 10^8^ CFU/g significantly increased FW, WG, SGR, FE, and FER of snakehead fish (*Channa argus*) and grass carp (*C. idella*), and SR of *Aeromonas hydrophila*-challenged grass carp showed the same trend [30,50]. Collectively, these studies confirm that *B. subtilis*, *C. butyricum*, and *E. faecalis* enhance feed utilization, growth performance, and stress resistance of fish.

### 4.2. Dietary Probiotics Increase Intestinal Digestive Enzyme and Antioxidant Enzyme Activity in Grass Carp

Numerous studies indicate that *Bacillus* and lactic acid bacteria can secrete exogenous enzymes and metabolites (e.g., short-chain fatty acids and vitamins) in the fish intestine, synergizing with endogenous enzymes to enhance nutrient digestion and absorption [16,20]. For example, supplementation of *C. butyricum* at 3 × 10^10^ to 3 × 10^11^ CFU/kg in Nile tilapia diets significantly increased trypsin, lipase, and amylase activities of fish [51]. In blunt snout bream (*Megalobrama amblycephala*), protease activity initially increased and then decreased with escalating *E. faecalis* supplementation (1 × 10^5^ to 1 × 10^7^ CFU/g), peaking at 1 × 10^6^ CFU/g [45]. Additionally, dietary *B. subtilis* (1.0 × 10^6^ to 1.0 × 10^10^ CFU/g) enhanced intestinal protease activity in Yellow River carp (*C. carpio* L.), Nile tilapia (*O. niloticus*), yellow catfish (*P. fulvidraco*), red sea bream (*Pagrus major*), striped catfish (*Pangasius hypophthalmus*), and olive flounder (*Paralichthys olivaceus*) [21,22,52,53,54]. Therefore, the positive effects of probiotics on intestinal digestive enzyme activity in fish have been well-documented [51,52,53,54]. Chymotrypsin, a serine endopeptidase, specifically hydrolyzes peptide bonds at aromatic amino acid residues (e.g., tryptophan, tyrosine, phenylalanine) [35]. Synthesized as inactive zymogens (chymotrypsinogen) in the pancreas, it is enzymatically activated upon intestinal secretion [35]. As a key determinant for protein metabolism and nutritional status [34,35,36,37,38], the increase of chymotrypsin activity has been reported in Caspian roach (*R*. *frisii kutum*) and grass carp (*C. idella*) fed with synbiotics or sodium butyrate [36,37]. This study showed upregulated expression of its precursor gene (*ctrb1*) in all probiotic-supplemented groups (*p* < 0.05), with the EF group exhibiting the highest *ctrb1* expression. This suggests that probiotics may enhance the intestinal digestive enzyme activity and protein metabolism by modulating the synthesis of chymotrypsin.

Antioxidant enzymes, including SOD and CAT, constitute the primary defense against oxidative stress. SOD catalyzes the conversion of O_2_^−^ to H_2_O_2_, which is further decomposed by CAT into H_2_O and O_2_ [55]. Dietary *C. butyricum* (1.5 × 10^6^–3.2 × 10^6^ CFU/g) significantly elevated hepatic SOD, CAT, and peroxidase (POD) activities in largemouth bass [49]. Similarly, *C. butyricum*-fermented feed (1000 g/kg) upregulated intestinal antioxidant enzyme activities (SOD, CAT, T-AOC) and related gene expression in grass carp [16]. Moreover, *B. subtilis* supplementation (1.0 × 10^6^–1.0 × 10^10^ CFU/g) enhanced SOD and CAT activities or regulated antioxidant genes (*nrf2*, *sod1*, *cat*) in grass carp (*C. idellus*), Japanese eel (*A. japonica*), olive flounder (*P. olivaceus*), rainbow trout (*Oncorhynchus mykiss*), and hybrid grouper (*Epinephelus coioides*) [40,46,54,56,57]. In this study, serum SOD and CAT activities were significantly elevated in the BS group (*p* < 0.05), while SOD activity increased notably in the CB group (*p* < 0.05). These improvements may stem from probiotic-induced modulation of gut microbiota and activation of the Nrf2-Keap1-ARE pathway, promoting antioxidant gene expression [16]. Although *E. faecalis* supplementation showed a non-significant upward trend in enzyme activities, this may reflect dose-dependent effects, as higher doses could attenuate its beneficial impact due to its conditional pathogenicity [58]. Overall, all three probiotics enhanced SOD activity, highlighting their critical role in mitigating oxidative stress in grass carp.

### 4.3. Dietary Probiotics Improve Intestinal Morphology and Microbial Community Composition in Grass Carp

Gut microbiota is a complex microbial community colonizing the fish gut, which plays a vital role in maintaining intestinal microecological balance. Numerous studies have demonstrated that dietary probiotics can enhance fish growth, lipid-glucose metabolism, and immune responses by modulating intestinal microbial diversity and composition [46,59]. Alpha-diversity indices reflect microbial community richness and diversity in fish intestines [46,59]. Specifically, the Ace and Chao indices represent community richness, which means higher values indicate greater species richness. The Shannon and Simpson indices reflect community diversity, integrating species richness and evenness. A higher Shannon index and lower Simpson index suggest greater diversity.

In this study, dietary supplementation with 1 × 10^10^ CFU/g of *B. subtilis*, *C. butyricum*, or *E. faecalis* significantly increased intestinal villus height in grass carp (*p* < 0.05). Moreover, *B. subtilis* notably elevated the intestinal V/C ratio (*p* < 0.05). Compared to the NC group, significantly lower Chao, Shannon, and Simpson indices were found in the BS and EF groups, while significantly higher Ace indices were found in the CB group (*p* < 0.05), indicating three probiotics had different impacts on the diversity of fish gut microbiota. These findings suggest that three probiotics supplements improved intestinal morphology, enhanced nutrient absorption, and had an obvious impact on the diversity of fish gut microbial communities. This phenomenon may arise from the dominance of specific beneficial taxa, which do not necessarily correlate positively with overall diversity, as previously reported in Chinese perch (*Siniperca chuatsi*) and large yellow croaker (*L. crocea*) [47,60].

**Figure 10:**
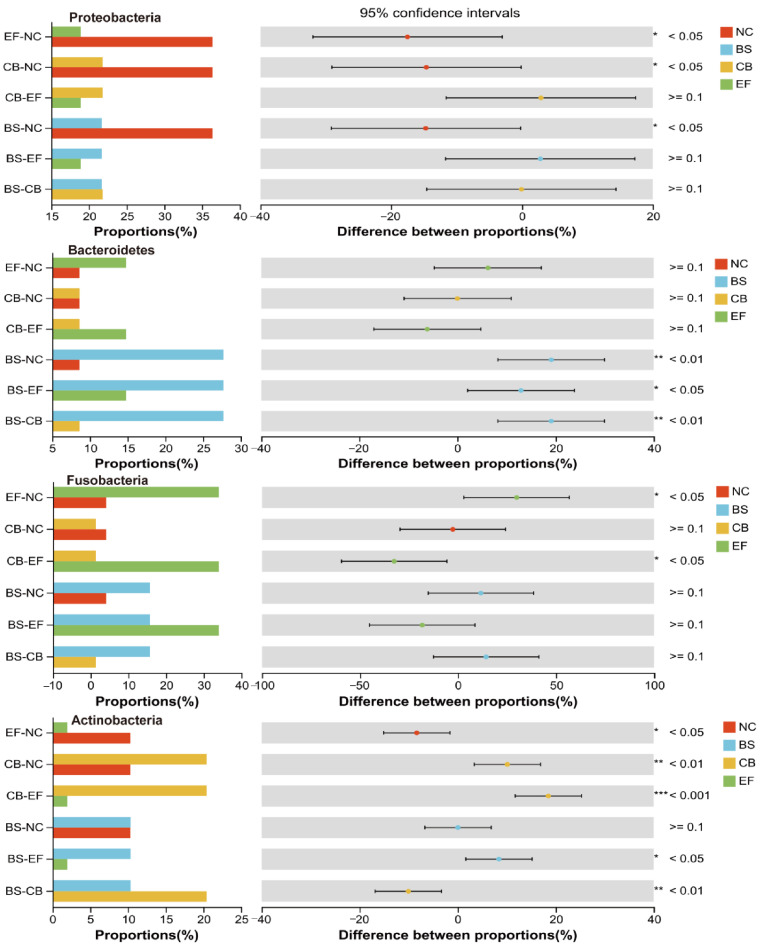
One-way ANOVA analysis of the comparison of gut microbial community on phylum level in different probiotic-supplemented groups and the control group. Values are means ± SE. Bars marked with * represent statistically significant differences (*p* < 0.05). Bars marked with ** represent statistically significant differences (*p* < 0.01). Bars marked with *** represent statistically significant differences (*p* < 0.001).

Composition, LEfSe, and RDA analyses of the gut microbiota of grass carp supplemented with different probiotics demonstrated that the relative abundance of Firmicutes was the highest in all groups at the phylum level, is 23.86%, 23.20%, 38.45%, and 28.34% in NC, BS, CB, and EF groups, respectively. The results indicated that the colonization of three probiotics in the fish gut was successful. Also, it was found that three probiotics significantly reduced the relative abundance of Proteobacteria (Figure 10) (*p* < 0.05), which is consistent with research on common carp and Chinese perch [61,62]. Proteobacteria are a Gram-negative bacterial phylum whose lipopolysaccharide-rich outer membrane can trigger intestinal inflammation [63]. The decrease in the abundance of this phylum may be attributable to the inhibitory effect of probiotic bacteria on the pathogenic bacteria within the intestinal tract [62].

In this study, the relative abundance of Actinobacteria in the intestinal tract was significantly higher in the *C. butyricum*-supplemented group compared to other groups (*p* < 0.05) (Figure 10). Actinobacteria are known to possess the capacity to impede the reproduction of potentially harmful bacteria through the production of antibiotic-active metabolites, thereby enhancing the immunity of fish [64]. The *B. subtilis*-supplemented group showed a significantly elevated relative abundance of Bacteroidetes compared to other groups (*p* < 0.05). The relative abundance of Actinobacteria was significantly increased after the supplementation of *C. butyricum* (1 × 10^10^ CFU/g) compared to the NC group (Figure 10) (*p* < 0.05). Also, the relative abundance of Fusobacteria was significantly higher in the *E. faecalis*-supplemented group than the NC group (Figure 10) (*p* < 0.05). Furthermore, the relative abundance of Actinobacteria and Fusobacteria was found to be positively correlated with the final body weight of grass carp in the study. Consistent with these findings, Sun et al. reported that supplementation of *E. faecium* L6 (1.0 × 10^8^ CFU/g) in diets significantly increased the relative abundance of Fusobacteria, Bacteroidetes, *Cetobacterium*, and *Bacteroides* levels in grass carp [44]. Bacteroidetes, Actinobacteria, and Fusobacteria have the capacity to degrade indigestible polysaccharides and generate short-chain fatty acids like butyrate, which could enhance the growth performance of fish by improving intestinal barrier function, ameliorating intestinal inflammation, and modulating gut microbiota homeostasis [64].

In this study, at the genus level, supplementation with 1 × 10^10^ CFU/g of *B. subtilis* significantly increased the relative abundance of *Prevotella_7*, *Neisseria*, *Leptotrichia*, and *Streptococcus* (*p* < 0.05). *Prevotella_7* could facilitate cellulose/hemicellulose digestion and SCFA production to improve lipid and glucose metabolism [65]. The *C. butyricum*-supplemented group showed increased abundance of *Lactobacillus* and *Clostridium_sensu_stricto_1* (*p* < 0.05), consistent with studies adding *B. subtilis* BOE9 or *E. faecium* AT5 to fish feed [62]. It has been reported that lactic acid bacteria (*Streptococcus* and *Lactobacillus*) could not only produce lactic acid to lower environmental pH but also produce antimicrobial compounds such as exopolysaccharides and H_2_O_2_, effectively inhibiting pathogen adhesion and colonization [66,67,68]. *Clostridium_sensu_stricto_1* could upregulate tight junction gene expression and suppress pro-inflammatory cytokine genes. The increased relative abundance of *Clostridium_sensu_stricto_1* in the *C. butyricum*-supplemented group suggests that probiotic supplementation improves intestinal microbiota balance to strengthen mucosal immunity, which is consistent with findings from Amoah et al. [69]. In this study, the supplementation with 1 × 10^10^ CFU/g of *E. faecalis* led to a substantial increase (*p* < 0.05) in the abundance of *Cetobacterium* and *Bacteroides*, and the abundance of them exhibited a positive correlation with the final body weight of the grass carp. *Bacteroides* has been shown to facilitate the digestion of polysaccharides in fish [30]. *Cetobacterium* has been confirmed to be capable of metabolizing and producing vitamin B_12_, which possesses antioxidant and anti-inflammatory properties that contribute to the alleviation of oxidative stress and the enhancement of immune function in fish [70,71].

In summary, this study reveals that dietary *B. subtilis*, *C. butyricum*, or *E. faecalis* supplementation could increase the relative abundance of Actinobacteria, Fusobacteria, Bacteroidota, *Lactobacillus*, *Cetobacteria*, *Clostridium_sensu_stricto_1*, and *Bacteroides* and decrease the relative abundance of gut-harmful bacteria Proteobacteria in grass carp. These results demonstrate that the addition of three probiotics could not only promote the colonization and growth of beneficial bacteria associated with short-chain fatty acid production, glycolipid metabolism, oxidative stress, and immune response, but also inhibit the adhesion of harmful bacteria in grass carp intestines. Therefore, three probiotics could improve the growth performance, antioxidant capacity, and intestinal structure in grass carp by regulating the balance of gut microbiota.

## 5. Conclusions

This study demonstrated that dietary supplementation with 1 × 10^10^ CFU/g of *B. subtilis*, *C. butyricum*, or *E. faecalis* significantly enhanced the FBW, WGR, SGR, and crude protein content in grass carp. Notably, three probiotic treatments increased serum SOD activity, with particularly significant improvements observed in the *B. subtilis*-supplement group. The probiotic supplementation significantly upregulated the relative expression level of the *ctrb1*, increased intestinal villus length, and modulated the gut microbiota composition by elevating the relative abundance of beneficial bacterial taxa while reducing the proportion of potentially harmful phyla such as Proteobacteria. In conclusion, the three probiotics improved growth performance and feed utilization in juvenile grass carp through multiple mechanisms, including upregulating the expression of the chymotrypsin gene to enhance digestive capacity, optimizing intestinal morphology, rebalancing gut microbiota composition, strengthening intestinal barrier function, and boosting antioxidant enzyme activity. These synergistic effects collectively contributed to increased survival rates and overall growth efficiency in the experimental subjects.

## Figures and Tables

**Table 1:** Feed formulation for grass carp in different probiotic-supplemented groups and the control group.

Ingredients (g/kg)	NC Group	BS Group	CB Group	EF Group
Fish meal	100.00	100.00	100.00	100.00
Soybean meal	270.00	270.00	270.00	270.00
Rapeseed meal	140.00	140.00	140.00	140.00
Cottonseed meal	140.00	140.00	140.00	140.00
Corn starch	200.00	200.00	200.00	200.00
Fish oil	20.00	20.00	20.00	20.00
Soybean oil	20.00	20.00	20.00	20.00
*B. subtilis* (200 × 10^8^ CFU/g)	0.00	0.50	0.00	0.00
*C. butyricum* (250 × 10^8^ CFU/g)	0.00	0.00	0.40	0.00
*E. faecalis* (50 × 10^8^ CFU/g)	0.00	0.00	0.00	2.00
Microcrystalline cellulose	48.50	48.00	48.10	46.50
Vitamin premix ^1^	10.00	10.00	10.00	10.00
Mineral premix ^2^	20.00	20.00	20.00	20.00
Calcium monobasic phosphate	15.00	15.00	15.00	15.00
Choline chloride (50%)	6.00	6.00	6.00	6.00
Cr_2_O_3_	5.00	5.00	5.00	5.00
Ethoxyquin (30%)	0.50	0.50	0.50	0.50
CMC-Na	5.00	5.00	5.00	5.00

Note: ^1^ Vitamin Premix Composition (per kg diet): Vitamin A, 2000 IU; Vitamin B_1_ (Thiamine), 5 mg; Vitamin B_2_ (Riboflavin), 5 mg; Vitamin B_6_, 5 mg; Vitamin B_12_, 0.025 mg; Vitamin D_3_, 1200 IU; Vitamin E (dl-α-tocopheryl acetate), 21 mg; Vitamin K_3_ (Menadione sodium bisulfite), 2.5 mg; Folic acid, 1.3 mg; Biotin, 0.05 mg; D-Pantothenic acid calcium salt, 20 mg; Inositol, 60 mg; Ascorbic acid (35% solution), 110 mg; Nicotinamide, 25 mg. ^2^ Mineral Premix Composition (per kg diet): MnSO_4_, 10 mg; MnSO_4_, 10 mg; KCl, 95 mg; NaCl, 165 mg; ZnSO_4_, 20 mg; KI, 1 mg; CuSO_4_, 12.5 mg; FeSO_4_, 105 mg; Na_2_SeO_3_, 0.1 mg; Cobalt, 1.5 mg.

**Table 2:** The growth performance parameters of grass carp in different probiotic-supplemented groups and the control group.

Parameters	NC Group	BS Group	CB Group	EF Group
SR (%)	55.00 ± 5.00 ^a^	61.67 ± 3.33 ^a^	85.00 ± 5.00 ^b^	78.33 ± 4.41 ^b^
IBW (g)	42.72 ± 1.13 ^a^	42.64 ± 1.36 ^a^	43.50 ± 0.50 ^a^	41.50 ± 0.58 ^a^
FBW (g)	48.17 ± 1.04 ^a^	53.70 ± 1.31 ^b^	65.07 ± 0.99 ^c^	65.38 ± 1.20 ^c^
WGR (%)	12.77 ± 0.58 ^a^	26.03 ± 1.17 ^b^	49.63 ± 2.86 ^c^	57.61 ± 4.00 ^c^
SGR (%/d)	0.43 ± 0.02 ^a^	0.83 ± 0.03 ^b^	1.44 ± 0.07 ^c^	1.62 ± 0.09 ^c^
FR (%/d)	0.91 ± 0.05 ^a^	1.69 ± 0.06 ^b^	1.69 ± 0.02 ^b^	1.78 ± 0.01 ^b^
FCR	2.14 ± 0.08 ^c^	1.74 ± 0.09 ^b^	1.40 ± 0.10 ^a^	1.38 ± 0.01 ^a^
PER	1.68 ± 0.06 ^a^	1.85 ± 0.22 ^a^	2.59 ± 0.18 ^b^	2.60 ± 0.03 ^b^

Note: Different letters in the same row indicate significant differences between groups (*p* < 0.05), while the same letters indicate no significant differences (*p* > 0.05).

**Table 3:** Intestinal morphology of grass carp in different probiotic-supplemented groups and the control group.

Parameters	NC Group	BS Group	CB Group	EF Group
VH (µm)	960.12 ± 25.59 ^a^	1103.38 ± 34.76 ^b^	1071.12 ± 19.31 ^b^	1047.15 ± 15.14 ^b^
CD (µm)	69.90 ± 1.43 ^a^	69.39 ± 0.89 ^a^	72.28 ± 0.31 ^a^	72.31 ± 0.86 ^a^
V/C	14.43 ± 0.34 ^a^	15.09 ± 0.50 ^b^	14.82 ± 0.27 ^ab^	14.48 ± 0.23 ^a^

Note: Values in the same row marked with different letters represent statistically significant differences (*p* < 0.05).

**Table 4:** Alpha diversity index of the intestinal microbiota of grass carp in different probiotic-supplemented groups and the control group.

Parameters	Groups			
	NC	BS	CB	EF
Chao	507.00 ± 28.30 ^b^	398.09 ± 32.43 ^a^	613.84 ± 30.09 ^c^	337.30 ± 8.94 ^a^
Ace	526.97 ± 49.01 ^ab^	625.26 ± 71.43 ^b^	583.20 ± 38.54 ^b^	376.40 ± 55.56 ^a^
Shannon	7.19 ± 0.34 ^b^	4.10 ± 0.00 ^a^	7.41 ± 0.09 ^b^	3.62 ± 0.04 ^a^
Simpson	0.98 ± 0.01 ^c^	0.91 ± 0.00 ^b^	0.99 ± 0.00 ^c^	0.79 ± 0.04 ^a^

Note: Values in the same row marked with different letters represent statistically significant differences (*p* < 0.05).

## Data Availability

The original contributions presented in this study are included in the article. Further inquiries can be directed to the corresponding authors.
